# Supplementary material for: Exploring the influence of self-identification on perceptual judgments of physical and social causality
Source: PeerJ. 2024 May 21;12:e17449. doi: 10.7717/peerj.17449 (PMC11122051; doi:10.7717/peerj.17449)
Supplement: Supplemental Information 1 — Separated analyses are presented for the matching task of Experiments 1 and 2. [file peerj-12-17449-s001.docx]

**Matching task analyses**

**Experiment 1**

Trials in which participants provided either a missed response (7.2 % of trials) or an incorrect response (20.8 % of trials) were removed and analysed separately. Correct responses with a RT lower than 200 ms (see also Sui, He & Humphreys, 2012) were considered anticipations and removed (1.24% of trials). Then, missed responses, incorrect responses, and RTs for correct responses were analysed through three different three-way repeated-measures ANOVAs with Shape category (2: self-related vs. stranger-related), Matching judgement (2: matched vs. nonmatching) and Block (2: first vs. second) as within-participant factors. The main results for incorrect responses and RTs for correct responses, averaged across the two blocks, are summarised in Figure S1.


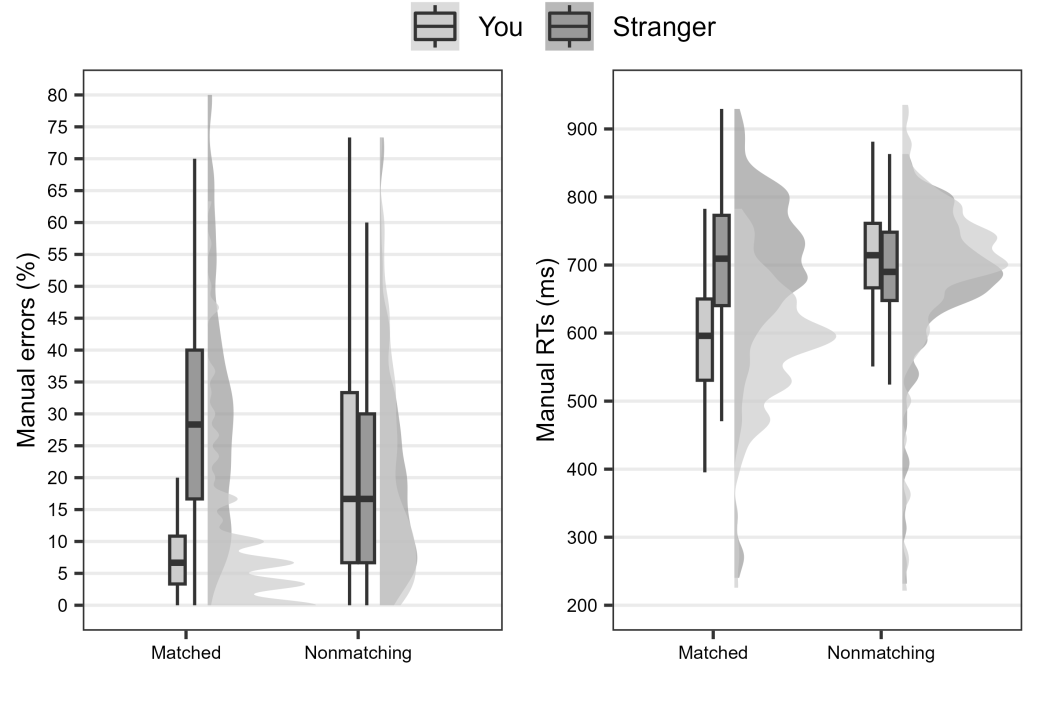


**Figure S1: Results observed in the matching task of Experiment 1.**

The lower and upper hinges of the boxplots correspond to the first and third quartiles of the distribution, the thick horizontal line corresponds to the median. The lower and upper whiskers extend from the lower and upper hinges to the lowest or largest value no further than 1.5 * IQR from the hinge, where IQR is the inter-quartile range. The density curves represent the distribution of the data.

As for incorrect responses, the main effect of Shape was significant [*F*(1, 58) = 117.01, *p* < .001, *η^2^_G_* = .102], due to fewer errors for the self-related shape (*M* = 15.48% , *SE* = 1.55) than for the stranger-related shape (*M* = 26.02% , *SE* = 1.47). The main effect of Block was also significant, [*F*(1, 58) = 59.85, *p* < .001, *η^2^_G_* = .078], since errors were higher in the first (*M* = 25.28% , *SE* = 1.57) than in the second (*M* = 16.21% , *SE* = 1.51) block. The Shape × Matching judgement interaction was significant [*F*(1, 58) = 59.38, *p* < .001, *η^2^_G_* = .109]. Post-hoc two-tailed *t*-tests with Bonferroni correction showed that, in Matched trials, fewer errors emerged for the self-related shape (*M* = 9.72% , *SE* = 1.44) than for the stranger-related shape (*M* = 31.16% , *SE* = 1.83; *t*(58) = -11.75, *p* < .001, *d* = -1.53) whereas the difference was non-significant in nonmatching trials [*t*(58) = 0.2, *p* > .10, *d* = .02]. The Shape × Block interaction was statistically significant [*F*(1, 58) = 5.19, *p* < .05, *η^2^_G_* = .004], and post-hoc two-tailed *t*-tests with Bonferroni correction showed that, in both blocks, fewer errors emerged for the self-related shape than for the stranger-related shape [*t*s < -7.0, *p*s < .001], although the difference was slightly larger in the first (*M* = 18.98% , *SE* = 1.86 vs. *M* = 31.58%, *SE* = 1.59) than in the second block (*M* = 11.98% , *SE* = 1.42 vs. *M* = 20.45% , *SE* = 1.82). The Shape × Matching judgement × Block interaction was also significant [*F*(1, 58) = 9.89, *p* < .005, *η^2^_G_* = .007] and two further ANOVAs were therefore conducted on each of the two Blocks with Shape and Matching judgements as within-participant factors. These analyses confirmed that, in both blocks, the main effect of Shape [*F*s > 49.85, *p*s < .001] and the Shape × Matching judgement interaction [*F*s > 36.39, *p*s < .001] were significant, whereas the main effect of Matching judgement was non-significant [*F*s < 1.3]. Hence, in both blocks, the self-prioritisation effect was present. No other significant results emerged [*F*s < 1.1, *p*s > .10].

As for latencies of correct responses, the main effect of Shape was significant [*F*(1, 58) = 78.63, *p* < .001, *η^2^_G_* = .044], due to smaller latencies for the self-related shape (*M* = 632 ms, *SE* = 9.91 ms) than for the stranger-related shape (*M* = 684 ms, *SE* = 11.5 ms). The main effect of Matching judgement was also significant [*F*(1, 58) = 121.73, *p* < .001, *η^2^_G_* = .053], due to smaller latencies in matched (*M* = 621 ms, *SE* = 10.02 ms) than in nonmatching (*M* = 691 ms, *SE* = 10.8 ms) trials. The main effect of Block approached the canonical level of significance [*F*(1, 58) = 3.25, *p* = .077, *η^2^_G_* = .012], suggesting that latencies tended to be smaller in the second (*M* = 642 ms, *SE* = 10.57 ms) than in the first (*M* = 664 ms, *SE* = 15.49 ms) block. The Shape × Matching judgement interaction was significant [*F*(1, 58) = 126.69, *p* < .001, *η^2^_G_* = .077]. Post-hoc two-tailed *t*-tests with Bonferroni correction showed that, in Matched trials, latencies were smaller for the self-related shape (*M* = 579 ms, *SE* = 9.64 ms) than for the stranger-related shape (*M* = 687 ms, *SE* = 13.4 ms; *t*(58) = -10.5, *p* < .001, *d* = -1.37). In nonmatching trials, the inverted pattern of results emerged, since latencies were smaller for the stranger-related shape (*M* = 685 ms, *SE* = 10.76 ms) than for the self-related shape (*M* = 699 ms, *SE* = 11.22; *t*(58) = 3.15, *p* = .015, *d* = .41). No other significant results emerged [*F*s < 1.3, *p*s > .1].

As for missed responses, the main effect of Shape was significant [*F*(1, 58) = 12.41, *p* = .006, *η^2^_G_* = .007], due to fewer missed responses for the self-related shape (*M* = 6.44% , *SE* = .91) than for the stranger-related shape (*M* = 7.95% , *SE* = 1.05). The main effect of Block was also significant [*F*(1, 58) = 48.73, *p* < .001, *η^2^_G_* = .1], since missed responses were higher in the first (*M* = 10.16% , *SE* = 1.31) than in the second block (*M* = 4.24% , *SE* =.7). The Shape × Matching judgement interaction was significant [*F*(1, 58) = 20.68, *p* < .001, *η^2^_G_* = .014]. Post-hoc two-tailed *t*-tests with Bonferroni correction showed that, in matched trials, fewer missed responses emerged for the self-related shape (*M* = 5.03% , *SE* =.81) than for the stranger-related shape (*M* = 8.73% , *SE* = 1.07; *t*(58) = -4.98, *p* < .001, *d* = .65) whereas the difference was non-significant in nonmatching trials [*t*(58) = 1.28, *p* > .10, *d* = .17]. The Shape × Block interaction was statistically significant [*F*(1, 58) = 8.77, *p* < .005, *η^2^_G_* = .003], and post-hoc two-tailed *t*-tests with Bonferroni correction showed that, in Block 1, fewer missed responses emerged for the self-related shape (*M* = 8.84% , *SE* = 1.26) than for the stranger-related shape (*M* = 11.47% , *SE* = 1.44; *t*(58) = -3.67, *p* < .005, *d* = -.48), whereas the difference was non-significant in Block 2 [*t*(58) = 1.05, *p* > .10, *d* = .14]. The Shape × Matching judgement × Block interaction was also significant *F*(1, 58) = 7.23, *p* < .01, *η^2^_G_* = .005] and two further ANOVAs were therefore conducted on each of the two Blocks with Shape and Matching judgements as within-participant factors. These analyses confirmed that, in both blocks, the Shape × Matching judgement interaction [*F*s > 4.2, *p*s < .04] was significant, suggesting that a self-prioritisation effect was present in both blocks. No other significant results emerged [*F*s < 2.0, *p*s > .10].

**Experiment 2**

Data were analysed as in Experiment 1. Trials in which participants provided either a missed response (4.7 % of trials) or an incorrect response (21.4 % of trials) were removed and analysed separately. Correct responses with a RT lower than 200 ms were considered outliers and removed (1.78% of trials). Then, missed responses, incorrect responses, and RTs for correct responses were analysed through three different three-way repeated-measures ANOVAs with Shape category (2: self-related vs. stranger-related), Matching judgement (2: matched vs. nonmatching) and Block (2: first vs. second) as within-participant factors. The main results for incorrect responses and RTs for correct responses, averaged across the two blocks, are summarised in Figure S2.


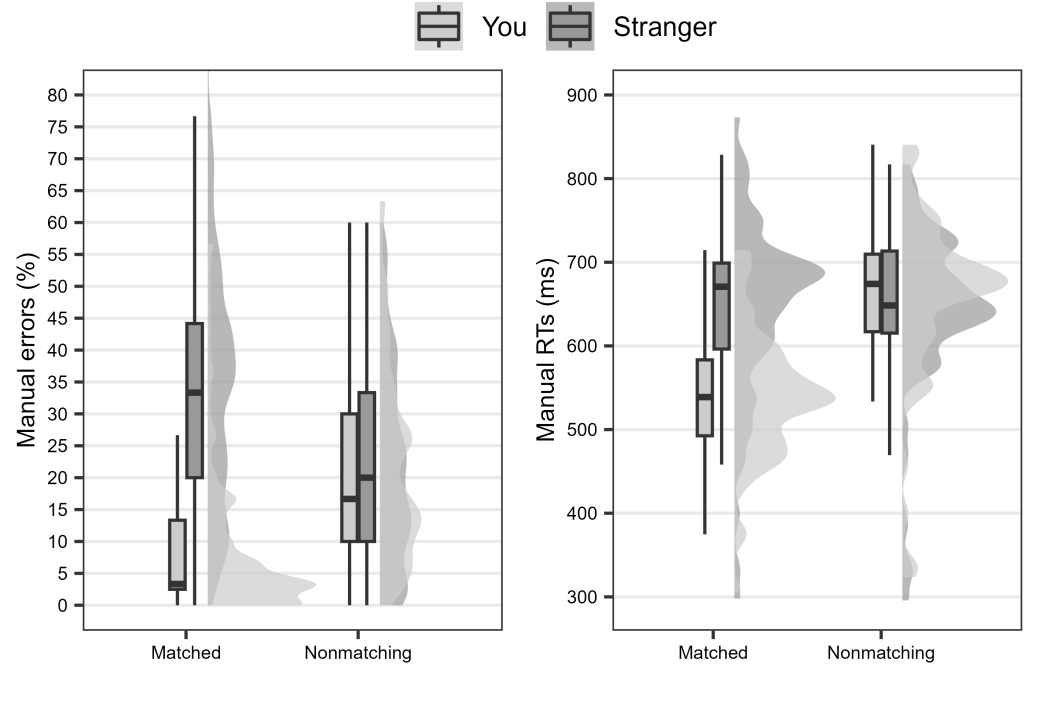


**Figure S2: Results observed in the matching task of Experiment 2.**

The lower and upper hinges of the boxplots correspond to the first and third quartiles of the distribution, the thick horizontal line corresponds to the median. The lower and upper whiskers extend from the lower and upper hinges to the lowest or largest value no further than 1.5 * IQR from the hinge, where IQR is the inter-quartile range. The density curves represent the distribution of the data.

As for incorrect responses, the main effect of Shape was significant [*F*(1, 61) = 127.59, *p* < .001, *η^2^_G_* = .152], due to fewer errors for the self-related shape (*M* = 15.13% , *SE* = 1.37) than for the stranger-related shape (*M* = 27.67% , *SE* = 1.5), as well as the main effect of block, [*F*(1, 61) = 45.82, *p* < .001, *η^2^_G_* = .056], due to fewer errors in the second (*M* = 17.78% , *SE* = 1.5) than in the first (*M* = 25.01% , *SE* = 1.35) block. The main effect of Matching judgement was non-significant [*F* < 1]. The Shape × Block interaction was significant, [*F*(1, 61) = 42.99, *p* < .001, *η^2^_G_* = .032], and post-hoc two-tailed *t*-tests with Bonferroni correction showed that, in both blocks, the self-related shape led to fewer error than the stranger-related shape [*t*s > 5.59, *p*s < .001], but the difference between self- and stranger-related shapes was greater in the first block (*M* = 17.88%, *SE* = 1.47) than in the second block (*M* = 7.18%, *SE* = 1.28). More importantly, the Shape × Matching judgement interaction was also significant, [*F*(1,61) = 110.26, *p* < .001, *η^2^_G_* = .142]. Post-hoc two-tailed *t*-tests with Bonferroni correction showed that, on matched trials, fewer errors emerged for the self-related shape (*M* = 9.0% , *SE* = 1.37) than for the stranger-related shape (*M* = 33.58% , *SE* = 1.95; *t*(61) = 12.43, *p* < .001, *d* = 1.58) whereas the difference was non-significant in nonmatching trials [*t*(61) = .44, *p* > .10, *d* = .006]. No other significant results emerged [*F*s < 1, *p*s > .10].

As for latencies of correct responses, the main effect of Shape was significant [*F*(1, 61) = 110.12, *p* < .001, *η^2^_G_* = .049], due to smaller latencies for the self-related shape (*M* = 593 ms, *SE* = 10.7 ms) than for the stranger-related shape (*M* = 645 ms, *SE* = 12.3 ms). The main effect of Matching judgement was also significant [*F*(1, 61) = 87.97, *p* < .001, *η^2^ _G_* = .056], due to smaller latencies on matched (*M* = 584 ms, *SE* = 10. 3 ms) than on nonmatching (*M* = 651 ms, *SE* = 12.7 ms) trials, as well as the main effect of Block [*F*(1, 61) = 20.8, *p* < .001, *η^2^ _G_* = .044], suggesting that latencies tended to be smaller in the second (*M* = 597 ms, *SE* = 10.7 ms) than in the first (*M* = 639 ms, *SE* = 13.9 ms) block. There was also a small but significant effect of the Shape × Block interaction [*F*(1, 61) = 4.39, *p* = .04, *η^2^_G_* = .001]. Post-hoc *t*-tests showed that, in both blocks, the latencies were smaller for the self-related shape than for the stranger-related shape [*t*s > 8.97, *p*s < .001], but the difference was greater in the first block (*M* = 64 ms, *SD* = 6.4 ms) than in the second block (*M* = 47 ms, *SD* = 5.2 ms). More importantly, the Shape × Matching judgement interaction was significant [*F*(1, 61) = 178.35, *p* < .001, *η^2^_G_* = .077]. Post-hoc two-tailed *t*-tests with Bonferroni correction showed that, in Matched trials, latencies were smaller for the self-related shape (*M* = 542 ms, *SE* = 9.7 ms) than for the stranger-related shape (*M* = 648 ms, *SE* = 13.1 ms; *t*(61) = 13.64, *p* < .001, *d* = 1.73). In nonmatching trials, the inverted pattern of results emerged, since latencies were slightly smaller for the stranger-related shape (*M* = 644 ms, *SE* = 12.6 ms) than for the self-related shape (*M* = 658 ms, *SE* = 13.3; *t*(61) = 3.16, *p* = .005, *d* = .4). No other significant results emerged [*F*s < 1, *p*s > .10].

As for missed responses, the main effect of Shape was not significant [*F*(1, 61) = 0.45, *p* > .10, *η^2^_G_* < .001]. The main effect of Matching judgement was significant, [*F*(1, 61) = 13.63, *p* < .01, *η^2^_p_* = .007], since missed responses were fewer on matched (*M* = 4.14% , *SE* = .62) than on nonmatching (*M* = 5.32% , *SE* =.78) trials, as well as the main effect of Block, [*F*(1, 61) = 43.96, *p* < .001, *η^2^_p_* = .131], since missed responses were fewer in the first (*M* = 7.46% , *SE* = 1.01) than in the second (*M* = 2.0% , *SE* = .38) block. The Shape × Matching judgement interaction was significant, [*F*(1, 61) = 15.13, *p* < .001, *η^2^_G_* = .013]. Post-hoc two-tailed *t*-tests with Bonferroni correction showed that, on matched trials, fewer missed responses emerged for the self-related shape (*M* = 3.41% , *SE* =.68) than for the stranger-related shape (*M* = 4.87% , *SE* =.67; *t*(61) = -2.77, *p* < .01, *d* = -.35) whereas the opposite pattern emerged on nonmatching trials, since fewer missed responses emerged for the stranger-related shape (*M* = 4.40% , *SE* =.75) than for the self-related shape (*M* = 6.24% , *SE* = .89; *t*(61) = -3.76, *p* < .001, *d* = -.48). The Matching judgement × Block interaction was significant, [*F*(1, 61) = 8.32, *p* < .01, *η^2^_G_* = .005]. Post-hoc two-tailed *t*-tests showed that, in the first block, fewer missed responses emerged for matched trials (*M* = 6.37% , *SE* = .96) than for nonmatching trials (*M* = 8.55%, *SE* =1.23; *t*(61) = -3.8, *p* < .001, *d* =.48), whereas no significant difference between matched trials (*M* = 1.91%, *SE* =.41) and nonmatching trials (*M* = 2.1%, *SE* = .42) emerged in the second block (*t*(61) = .56, *p* > .10, *d* =.07). The Shape × Matching judgement × Block interaction was also significant, [*F*(1, 61) = 11.77, *p* < .005, *η^2^_G_* = .008], and two further ANOVAs were therefore conducted on each of the two Blocks with Shape and Matching judgements as within-participant factors. These analyses showed that, in the first block, the main effect of Matching judgement and the Shape × Matching judgement interaction were both significant, [*F*s > 14.43, *p*s < .001] and the main effect of Shape was non-significant [*F* =.71, *p* > .10], whereas, in the second block, only non-significant results emerged, [*F*s < 1.57, *p*s > .10].
